# Fetal-Type Variants of the Posterior Cerebral Artery and Concurrent Infarction in the Major Arterial Territories of the Cerebral Hemisphere

**DOI:** 10.1177/2324709616665409

**Published:** 2016-09-13

**Authors:** Stephen L. Lambert, Frank J. Williams, Zhora Z. Oganisyan, Lionel A. Branch, Edward C. Mader

**Affiliations:** 1 Louisiana State University School of Medicine, New Orleans, LA, USA; 2 Louisiana State University Health Sciences Center, New Orleans, LA, USA

**Keywords:** stroke, posterior cerebral artery, fetal variant, developmental, angiography

## Abstract

Fetal-type or fetal posterior cerebral artery (FPCA) is a variant of cerebrovascular anatomy in which the distal posterior cerebral artery (PCA) territory is perfused by a branch of the internal carotid artery (ICA). In the presence of FPCA, thromboembolism in the anterior circulation may result in paradoxical PCA territory infarction with or without concomitant infarction in the territories of the middle (MCA) or the anterior (ACA) cerebral artery. We describe 2 cases of FPCA and concurrent acute infarction in the PCA and ICA territories—right PCA and MCA in Patient 1 and left PCA, MCA, and ACA in Patient 2. Noninvasive angiography detected a left FPCA in both patients. While FPCA was clearly the mechanism of paradoxical infarction in Patient 2, it turned out to be an incidental finding in Patient 1 when evidence of a classic right PCA was uncovered from an old computed tomography scan image. Differences in anatomical details of the FPCA in each patient suggest that the 2 FPCAs are developmentally different. The FPCA of Patient 1 appeared to be an extension of the embryonic left posterior communicating artery (PcomA). Patient 2 had 2 PCAs on the left (PCA duplication), classic bilateral PCAs, and PcomAs, and absent left anterior choroidal artery (AchoA), suggesting developmental AchoA-to-FPCA transformation on the left. These 2 cases underscore the variable anatomy, clinical significance, and embryological origins of FPCA variants.

## Introduction

Posterior cerebral artery (PCA) cortical branches supply blood to the occipital lobe, the inferomedial temporal lobe, and portions of the posterior inferior parietal lobe.^1^ Most adult humans have the classic vascular anatomy in which both left and right PCAs originate from the basilar artery and are part of the vertebrobasilar system or posterior circulation.^2^ An anatomic variant of the PCA, known as fetal-type or fetal PCA (FPCA), has been detected by anatomic^3-6^ and angiographic^7-10^ studies in 11% to 46% of adult humans, either unilaterally or bilaterally. Differences in detection method and definition may account for the variance in reported prevalence. In the definition proposed by van Raamt et al, an FPCA is called a *full FPCA* if the P1 segment is not visualized on computed tomography angiography (CTA), magnetic resonance angiography (MRA), or after injection of contrast into the vertebral artery; a *partial FPCA* if the P1 segment is smaller than the posterior communicating artery (PcomA); or an *intermediate FPCA* if the P1 segment is as large as the PcomA.^11^ When present, the FPCA supplies blood to parts of the cerebrum, which, in the majority of humans, are perfused by distal PCA branches.^7,8^ Like the middle cerebral artery (MCA) and the anterior cerebral artery (ACA), the FPCA is a branch of the internal carotid artery (ICA) and is therefore a part of the carotid system or anterior circulation.

Although the FPCA is considered a normal anatomic variant, its presence may modify the distribution and severity of cerebral injury from thromboembolic events. A few patients with acute ischemic stroke and simultaneous or nearly simultaneous PCA-ICA territory infarction were found to have an FPCA ipsilateral to the paradoxical PCA occlusion.^12-15^ Concurrent infarction occurred in the PCA and MCA territories of one hemisphere,^12-14^ except in one case where infarction occurred in the PCA, MCA, and ACA territories of the right hemisphere.^15^ We report 2 cases of concurrent ICA-PCA territory infarction in the setting of a unilateral FPCA. An FPCA was clearly visible on the left in both patients. Infarction occurred in the right MCA and PCA territories in Patient 1 and in the left ACA, MCA, and PCA territories in Patient 2.

## Case Presentation

Both patients are 82-year-old right-handed women, both had a large ischemic stroke involving the anterior and posterior circulation territories of one hemisphere, and both were found to have an FPCA. All of these coincidences can be confusing, so we must point out that the patients were not related and that one patient had a stroke several months before the other. In the emergency department, both patients had focal neurological deficits and head CT did not show hemorrhage. Neither patient qualified for tissue plasminogen activator therapy because of the extent of cerebral injury and the presence of at least one more contraindication: the absence of a clear time of symptom onset in Patient 1 and ongoing warfarin therapy in Patient 2 (prothrombin time = 21.3, international normalized ratio = 1.9).

**Figure 1.**
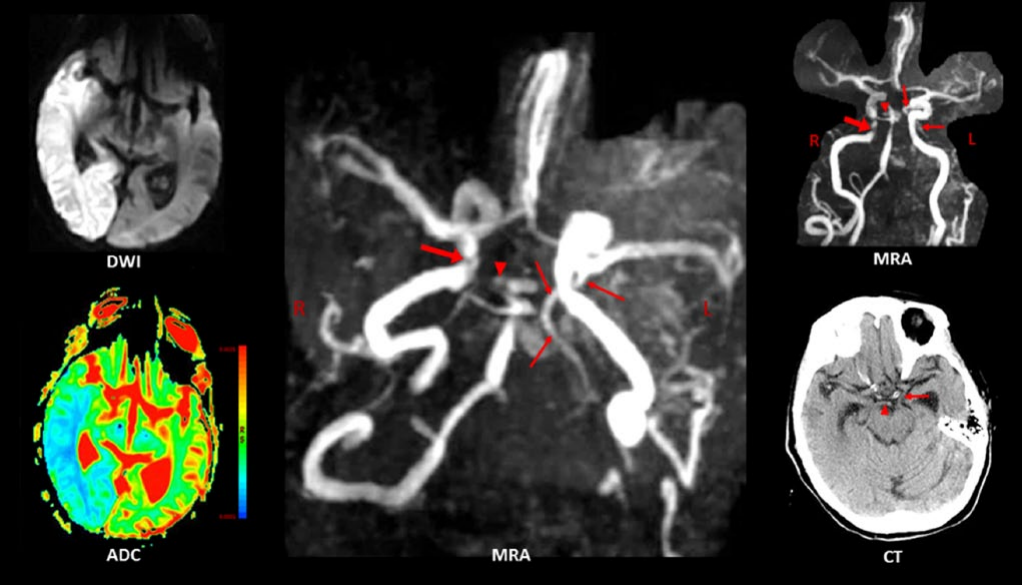
Patient 1 had acute infarcts in the right MCA and PCA territories (hyperintense DWI, low ADC). The 3D time-of-flight (TOF) MRA images show a left FPCA (long arrow), a remnant of the P1 segment (arrowhead) of the right PCA (the rest of it is absent), stenosis at the petrous segment of the right ICA (short arrow), stenosis of the basilar artery above and below the SCA origin (only right SCA is visible), and absent flow in the left vertebral artery. The CT scan image shown was taken 2½ years prior to admission and retrieved from her old records. It clearly shows an intact classic right PCA (arrowhead) and a left fetal-type PCA (long arrow).

Patient 1 had myocardial infarction, atrial fibrillation, and mitral valve replacement with porcine valve in the past. She was on warfarin in the past but this was stopped due to diverticulosis and replaced with aspirin. Considering she has mild dementia, her ability to comply is questionable. She had an infarction in the cerebellum 2 months prior to admission from which she fully recovered. On arrival to the emergency room, her vital signs were as follows: blood pressure (BP) = 152/71, heart rate (HR) = 80 (regular), respiratory rate (RR) = 22, and temperature = 36.8°C. She was somnolent and responded to questions by moaning, otherwise she was able to comprehend and execute simple commands. Focal findings consisted of left spatial neglect, preferential gaze to the right, inability to track toward the left, upper motor neuron pattern of left face weakness, distal > proximal pattern of weakness of the left upper (1/5), and left lower extremities (2/5), absence of deep tendon reflexes on the left, and a left Babinski sign (National Institutes of Health Stroke Scale [NIHSS] score = 27). Brain MRI on the second hospital day showed acute infarcts in the right MCA and PCA territories (Figure 1: diffusion-weighted imaging [DWI] and apparent diffusion coefficient [ADC]). MRA revealed a left FPCA, a remnant of the P1 segment of the right PCA, stenosis at the petrous segment of the right ICA, stenosis of the basilar artery above and below the SCA origin, and absent flow in the left vertebral artery (Figure 1: MRA). Because the FPCA was contralateral to the infarcted hemisphere, we hypothesized the presence of FPCAs bilaterally with the right FPCA invisible because it was occluded. However, an old CT image (taken 2½ years prior to admission and retrieved from her old records) revealed a classic right PCA (Figure 1: CT). Electrocardiography showed normal sinus rhythm, and echocardiography revealed moderate mitral and aortic regurgitation. Because of cerebral edema, her sensorium declined over the first few days and eventually stabilized. Unfortunately, she did not regain full alertness and her deficits did not improve despite early physiotherapy in the acute care setting. After 3 weeks, she was transferred to a long-term care facility.

**Figure 2.**
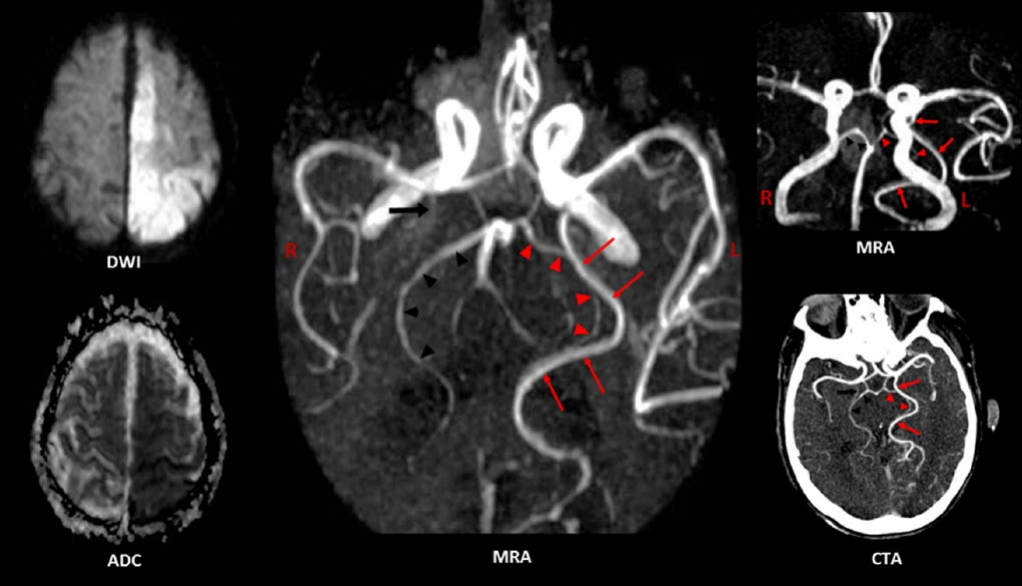
Patient 2 had acute infarcts in the left ACA, MCA, and PCA territories (hyperintense DWI, low ADC). The 3D time-of-flight (TOF) MRA images show 2 PCAs on the left (PCA duplication)—a dominant fetal-type PCA (arrows) and a small-caliber classic PCA (arrowheads). The left PCA and the right PCA (dark arrowheads) branch off the basilar artery. Two posterior communicating arteries and a right anterior choroidal artery (large dark arrow) are also present (middle MRA and CTA images). There is no evidence of flow-disrupting stenosis.

Patient 2 had 3 major stroke risk factors—hypertension, hyperlipidemia, and atrial fibrillation—for which she takes warfarin. Before the stroke, she lived in an assisted living facility and was able to drive a car. She was preparing a meal when another person found her slouched over a drawer. Emergency responders noted her to be somnolent, rousing occasionally to painful stimulation, but without speech output. On arrival to the emergency room, her vital signs were as follows: BP = 167/82, HR = 85/min (irregular), RR = 20, and temperature = 36.7°C. Her neurological examination was remarkable for profound impairment of language function involving comprehension and expression, distal > proximal pattern of weakness of the right upper (2/5) and right lower (1/5) extremities, and a right Babinski sign (NIHSS score = 31). It was impossible to assess her aphasia amid her depressed sensorium, the latter most likely due to cerebral edema and mass effect from the large stroke. Brain MRA on the second hospital day showed acute infarcts in the left ACA, MCA, and PCA territories (Figure 2: DWI and ADC). MRA and CTA did not show any flow-disrupting stenosis but an anatomic variant, known as PCA duplication, was present on the left—the dominant PCA was an FPCA and the smaller PCA was a classic PCA (Figure 2: MRA and CTA). Other findings include bilateral classic PCAs and posterior communicating arteries (PcomAs) and an anterior choroidal artery (AchoA) on the right but not on the left (Figure 2: middle MRA and CTA). Electrocardiography showed atrial fibrillation and echocardiography revealed biatrial enlargement. Her mental status declined over a few days due to worsening cerebral edema. An ictal contribution to the depressed sensorium was confirmed when electroencephalography showed intermittent epileptiform activity in the left temporal region. Intravenous levetiracetam was administered but it was the regression of cerebral edema that correlated with the resolution of cortical hyperexcitability. Because of the size of the infarct, warfarin was temporarily placed on hold. She became slightly more arousable but neither her language function nor her strength on the right showed signs of improvement. After 12 days, she was discharged to a skilled nursing facility.

## Discussion

Both patients had acute ischemic strokes (high DWI signal intensity with corresponding low ADC) and concurrent infarction (simultaneous occurrence of symptoms and similar DWI signal intensities) in the ICA and PCA territories of one hemisphere (Figures 1 and 2: DWI and ADC images). Noninvasive cerebral angiography detected a left FPCA in both patients (Figure 1: MRA images; Figure 2: MRA and CTA images). However, the clinical significance and developmental implications of FPCA in each patient are quite different.

Emboli can move up the ICA, enter and occlude the FPCA or its branches, and result in a paradoxical PCA territory infarction—with^12-15^ or without^16-20^ attendant occlusion of other ICA branches. A left FPCA was clearly the reason for the left-sided paradoxical infarction in Patient 2. On the other hand, the reason for the right-sided paradoxical infarction in Patient 1 was not obvious. MRA showed high-grade stenosis of the right ICA petrous segment (see Figure 1 for complete MRA findings) indicating that right ICA thrombosis and embolism resulted in right MCA and PCA territory infarction, the latter via a right FPCA. However, MRA showed a left FPCA and absent right PCA (except for a P1 remnant). As there was no concrete evidence of a right FPCA, we argued—as Eswaradass et al did in their case of concurrent ICA and PCA territory infarction^15^—that an FPCA was present ipsilateral to the infarct but was angiographically invisible because it was occluded. This argument was proved wrong when we discovered evidence of a classic right PCA in the patient’s old CT scan files (Figure 1: CT). All things considered, the most likely mechanism of paradoxical PCA occlusion in Patient 1 is right ICA-to-PCA embolism through a patent PcomA. Although an FPCA has been implicated in nearly all cases of paradoxical PCA occlusion,^12-20^ the PcomA is a potential conduit for cross embolization from the ICA to the PCA P2 segment or its distal branches.^12,21^ The rarity of PcomA embolism may be due to the low ICA-PCA pressure gradient across the PcomA.^12^ Factors that increase the ICA-PCA pressure gradient, such as hemodynamic changes from stenotic lesions in the vertebrobasilar system or the site of ICA thrombosis relative to the PcomA orifice, may increase the risk of PcomA embolism.

Cardioembolism with concomitant PCA and MCA branch occlusion is a plausible mechanism of PCA-MCA territory infarction in Patient 1, especially since she had atrial fibrillation in the past. However, she was in normal sinus rhythm throughout her hospital stay and echocardiography did not show any embolic source (albeit a transesophageal study was not performed). The study of Yang et al also argue against a cardioembolic mechanism in Patient 1.^12^ In this study, simultaneous ipsilateral anterior and posterior circulation territory infarction was present in only 21 (1.5%) of 1388 acute stroke patients. Of the 21 patients, 16 had angiographic evidence of ICA stenosis ipsilateral to the infarct and only one suffered cardioembolism. All but one patient with concurrent PCA-MCA infarction and an FPCA or a patent PcomA had significant ICA stenosis ipsilateral to the infarct.^12^ In earlier reports of FPCA and concurrent PCA-ICA infarction, only the MCA (not the ACA) territory of the ICA was involved.^12-14^ Recently, Eswaradass et al reported the first case of simultaneous PCA, MCA, and ACA territory infarction in association with a possible FPCA; MRA showed complete occlusion of the right ICA and a right FPCA was inferred based on the absence of the P1 segment on MRA.^15^ Patient 2 also had ACA, MCA, and PCA territory infarction but, unlike the case of Eswaradass et al, a definite FPCA was found (along with a classic PCA) ipsilateral to the stroke (Figure 2: MRA and CTA). In the absence of ICA stenosis and in the presence of atrial fibrillation, the 3-territory stroke in Patient 2 is most likely the result of cardioembolism to the right ACA, MCA, and FPCA (all 3 vessels are branches of the right ICA).

**Figure 3.**
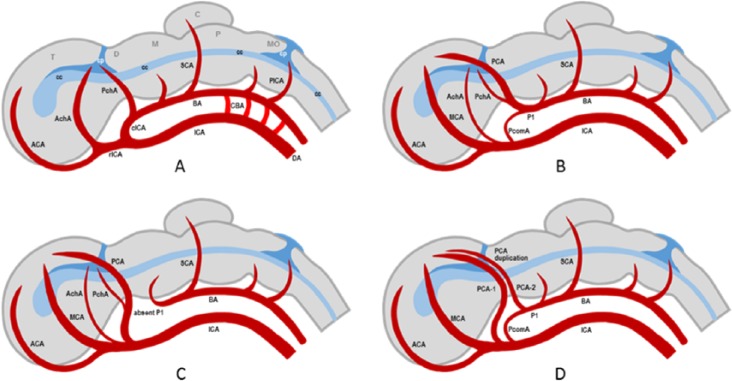
Diagram of human embryo in the choroidal stage (~5 weeks gestational age); only the left side is shown. (A) Some of the primitive cerebral arteries that are present during this stage are shown. The internal carotid artery (ICA) bifurcates into a rostral branch (rICA) and a caudal branch (cICA); the latter is the precursor of the posterior communicating artery (PcomA). The anterior cerebral artery (ACA) is a primitive rICA branch. Two large choroidal arteries are present: the anterior choroidal artery (AchoA), a branch of the rICA, and the posterior choroidal artery (PchoA), a branch of the cICA. Also shown are the precursors of the superior cerebellar artery (SCA) and posterior inferior cerebellar artery (PICA). Note that the anterior circulation supplies blood to the telencephalon (T), diencephalon (D), midbrain (M), pons (P), cerebellum (C), and medulla oblongata (MO). (B) Later-stage arteries (approaching the adult pattern) are superimposed on the same choroidal-stage embryo to show how they evolve. The middle cerebral artery (MCA) and posterior cerebral artery (PCA) become dominant and the choroidal arteries and PcomA regress but do not disappear. (C) In the most common variant of the fetal-type PCA, the PCA is a continuation of the PcomA and the P1 segment of the PCA (P1) regresses and may or may not disappear (absent in the diagram). This is the case in Patient 1. (D) In the less common variant of the fetal-type PCA, there are 2 PCAs (PCA duplication)—one is derived from the AchoA (often dominant) and the other is a classic PCA (often small). This is the case in Patient 2. BA, basilar artery; D, dorsal aorta; CBA, carotid-basilar anastomoses; cp, choroid plexus; cc, central canal (later develops into the ventricular system).

The label “fetal PCA” has been attached to a number of developmental variants of the adult cerebral arterial system where a significant portion of the distal PCA territory is perfused through a branch of the ICA. However, experts do not always agree on whether or not a particular variant should be called “fetal PCA.”^22^ The new nomenclature of PCA variants proposed by Masoud et al avoids the term “fetal PCA.”^23^ Some authors emphasize the importance of distinguishing PCA variants that are “truly fetal” from those that are not.^24^ Different PCA variants can only be properly understood in the context of vascular neuroembryology.^25^ The reader whose field entails a deep understanding of vascular neuroembryology can benefit immensely from the website http://neuroangio.org/^22,25^ and from excellent publications on this subject, some of which are included in our bibliography.^26-33^ We also created highly simplified and idealized diagrams to help the reader acquire a basic idea of cerebrovascular embryology (Figure 3).

Figure 3 is a schematic of the developmental changes in the cerebral arterial circulation during human embryogenesis: only pertinent cerebral arteries are shown. Figure 3A shows the embryo in the choroidal stage with a gestational age of about 5 weeks.^25,26^ The anterior circulation (ICA and its branches) perfuses the cerebrum and curves back to supply the upper brainstem, while carotid-basilar anastomoses (CBAs) supply the lower brainstem.^27-30^ The terminal ICA is bifurcated into a rostral (rICA) and a caudal (cICA) division.^25,30^ The main rICA branches are the ACA and the AchoA. The cICA is the future PcomA and its main branches are the PchoA and the future P1 segment of the PCA.^29,30^ The superior cerebellar artery (SCA) is the only major artery supplying the primitive cerebellum during this stage.^25,27^ As the posterior cerebrum, cerebellum, and brainstem grow and the ICA can no longer keep up with their metabolic demands, the posterior circulation develops from the primitive arterial mesh at an accelerated pace.^29,30^ The posterior circulation increasingly becomes independent of the anterior circulation and the CBAs regress and disappear, except in a few individuals where a CBA may persist as an anatomic variant, for example, persistent trigeminal artery.^31^ Figure 3B, C, and D illustrates 3 future trajectories of angiogenesis beyond the choroidal stage. Other outcomes are possible but only these 3 patterns are relevant to our discussion. Figure 3B shows how the typical PCA pattern is achieved. The MCA and PCA take over the dominant roles of the AchoA and PchoA, respectively. Both choroidal arteries regress but usually do not disappear. Part of the PchoA is annexed by the evolving PCA. The cICA regresses becoming the PcomA. Figure 3C shows how the common FPCA variant develops. The cICA (PcomA) continues to be dominant and becomes the proximal FPCA segment that connects with the ICA. The P1 segment of the PCA regresses and may disappear (absent in diagram). This is the case in Patient 1. Figure 3D shows how the “true fetal” PCA variant develops. Two PCAs emerge (PCA duplication)—one PCA is derived from the AchoA (often dominant) and the other PCA is a classic PCA (often smaller). This is the case in Patient 2. Some authors refer to this anatomic variant, not as FPCA or “true fetal” PCA, but as hyperplastic AChA.^24,32,33^

With a prevalence rate in the 11% to 46% range^3-10^ and an established role in the pathogenesis of multi-territory or paradoxical strokes,^12-21^ FPCA certainly deserves more clinical attention. FPCA should be suspected in patients with infarcts in the anterior and posterior circulation territories of one hemisphere. Currently, the fastest and safest method for detecting FPCA is MRA or CTA. High-grade carotid stenosis and FPCA ipsilateral to the infarcted hemisphere may obviate additional workup in search of a cardioembolic source. If the stroke cannot be explained by carotid disease, the likelihood of cardioembolism is high; transesophageal echocardiography, mobile cardiac outpatient telemetry, or loop recorder should be considered if transthoracic echocardiography or Holter monitoring are unrevealing. By being aware that an FPCA exists, surgeons can plan their surgical approach or endovascular procedure to minimize the risk of perioperative cerebral hypoperfusion or embolization.^34,35^ Preoperative vascular imaging and assessment of collateral blood flow is important in planning carotid endarterectomy or stenting.^36^ FPCA ipsilateral to the stenotic ICA is a compelling reason to use embolic protection devices and to implement intraoperative monitoring to prevent cerebral hypoperfusion during carotid endarterectomy. Failure to recognize an FPCA can also lead to errors in interpreting cerebral perfusion studies. Wentland et al found that a unilateral FPCA can affect perfusion measurements and give rise to perfusion map asymmetries.^37^

Arguably, the presence of FPCA in individuals with major risk factors for cerebral embolism (eg, ICA stenosis, atrial fibrillation) should encourage physicians to be more aggressive in addressing these risk factors. Kolukısa et al reported a case of sequential infarction (first left PCA, then left MCA territory) in the setting of left FPCA and extracranial ICA stenosis; stabilization of cerebral perfusion was achieved by carotid endarterectomy.^14^ Albeit intuitively appealing, there is no solid evidence in favor of more aggressive antiplatelet or anticoagulant regimens in individuals with FPCA and major stroke risk factors. It is also not clear whether FPCA increases the overall risk of stroke independent of other risk factors. Yang et al found a “causative” FPCA ipsilateral to the infarct in 44.4% of acute stroke patients but only 18.5% of individuals in their control group have “incidental” FPCA.^12^ Two autopsy studies found more FPCAs in brains with infarcts than in infarct-free brains.^38,39^ According to van Raamt et al, a full FPCA carries a higher vascular insufficiency risk than a partial FPCA because leptomeningeal anastomoses do not form between the anterior and posterior circulation if a person has a full FPCA.^11^ After reviewing the literature, Brozici et al realized the wide range of individual variability in the size, distribution, and number of leptomeningeal anastomoses; the authors pointed out that studies addressing the range of variability or that link the variability to compensatory capacity are lacking.^40^ Clinical studies have had mixed results: increased risk of cerebral ischemia from FPCA,^41^ enhanced risk only with full FPCA,^42^ greater risk with partial FPCA,^43^ and no increase in stroke risk^44^ have all been observed. Whether FPCA increases the overall risk of stroke independent of other risk factors is still an open question.

## Conclusion

These 2 cases of fetal PCA (FPCA) and concurrent PCA-ICA territory infarction illustrate the variability in anatomy, clinical significance, and embryological origin of PCA variants. FPCA is clearly the mechanism of paradoxical infarction in one patient. In the other patient, FPCA is only an incidental finding, with an old CT scan image (not an angiogram) serving as confirmatory evidence. Differences in angiographic anatomy indicate that the FPCAs of the 2 patients are developmentally distinct—the FPCA of one patient appears to be an extension of the embryonic left posterior communicating artery, while the FPCA of the other patient is most likely derived from an embryonic anterior choroidal artery.

FPCA can increase the extent and severity of anterior circulation strokes by allowing additional infarction in the PCA territory. The physician should therefore vigorously address the stroke risk factors of individuals with FPCA, such as ICA stenosis and atrial fibrillation. Whether FPCA increases the overall risk of stroke independent of other risk factors is not clear. The optimal stroke prevention regimen for individuals with FPCA and one or more stroke risk factors is also unclear. With increasing accessibility to noninvasive neurovascular imaging, it will not be long before clinical researchers find solutions to these problems.
